# Phytophagous Insects on Native and Non-Native Host Plants: Combining the Community Approach and the Biogeographical Approach

**DOI:** 10.1371/journal.pone.0125607

**Published:** 2015-05-08

**Authors:** Kim Meijer, Hidde Zemel, Satoshi Chiba, Christian Smit, Leo W. Beukeboom, Menno Schilthuizen

**Affiliations:** 1 Groningen Institute of Evolutionary Life Sciences (GELIFES), University of Groningen, Groningen, the Netherlands; 2 Altenburg & Wymenga Ecological Consultants, Veenwouden, the Netherlands; 3 Ecology and Evolutionary Biology, Graduate School of Life Sciences, Tohoku University, Aobayama, Sendai, Japan; 4 Naturalis Biodiversity Center, Darwinweg 2, Leiden, the Netherlands; University of Heidelberg, GERMANY

## Abstract

During the past centuries, humans have introduced many plant species in areas where they do not naturally occur. Some of these species establish populations and in some cases become invasive, causing economic and ecological damage. Which factors determine the success of non-native plants is still incompletely understood, but the absence of natural enemies in the invaded area (Enemy Release Hypothesis; ERH) is one of the most popular explanations. One of the predictions of the ERH, a reduced herbivore load on non-native plants compared with native ones, has been repeatedly tested. However, many studies have either used a community approach (sampling from native and non-native species in the same community) or a biogeographical approach (sampling from the same plant species in areas where it is native and where it is non-native). Either method can sometimes lead to inconclusive results. To resolve this, we here add to the small number of studies that combine both approaches. We do so in a single study of insect herbivory on 47 woody plant species (trees, shrubs, and vines) in the Netherlands and Japan. We find higher herbivore diversity, higher herbivore load and more herbivory on native plants than on non-native plants, generating support for the enemy release hypothesis.

## Introduction

With the increase of human population density, colonisation opportunities for plants have changed dramatically. Many plant species have been transported, either intentionally or accidentally, outside their native habitat and imported into novel areas. In most countries at least 10% of the flora is introduced, ranging up to almost 50% in some areas, like New Zealand [1]. Often, these introductions lead to ecological damage. For example, in Europe, several species of non-native knotweed (*Fallopia* spp.) negatively influence the abundance and diversity of native plant and insect species [2]. In many cases, economic damage comes in the wake of such ecological upheaval [3].

Although the ecology of non-native species has been studied for many decades, it remains poorly understood why certain non-native species spread rapidly and become invasive [4–9], whereas most others do not. One factor that has frequently been suggested to play a major role in non-native species’ success is the fact that species in a novel habitat or area may suffer less from natural enemies [10,11]. This is known as the enemy release hypothesis (ERH).

The basic prediction of the ERH, namely that species in their non-native area are relatively free from parasites and predators, has been tested frequently. However, these tests have often been conducted with rather small numbers of plant species (<10) and have sometimes met with contradictory results [6,8,9,12].

**Fig 1:**
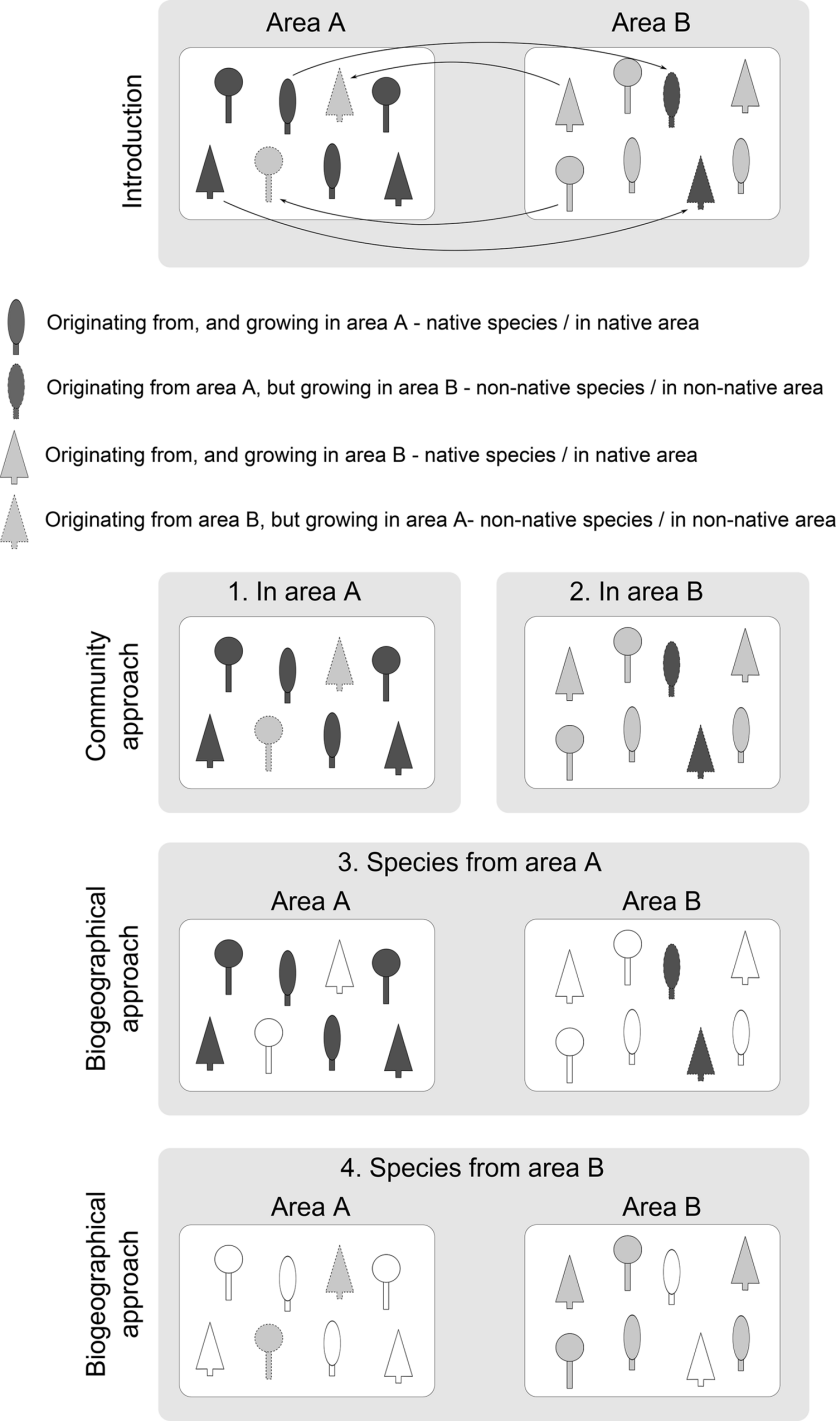
Measures of enemy release can be compared between native and non-native species using either the community or the biogeographical approach. The community approach compares native and non-native species within the same area. The biogeographical approach compares the same species in their native as well as in the non-native area. If species have been introduced from habitat A to B and vice versa both approaches can be used reciprocally. In this figure species originating from area A are dark grey, from area B light grey.

Most of these studies were performed using the community approach (Fig 1), where native and non-native species were studied within the same area (community). The advantage of studying species within the same area is that environmental factors are kept constant. The disadvantage is that the native species are likely to be phylogenetically unrelated to the non-native species that are being studied. Therefore, in studies with relatively low numbers of species considered, the results may be strongly affected by the plant taxa involved. Kennedy and Southwood [13], for example, found phytophagous insect species richness covering two orders of magnitude in a number of native plants: from *Taxus baccata* (6 spp.) and *Ilex aquifolium* (10 spp.) to *Crataegus monogyna* (209 spp.) and *Quercus petraea* + *robur* (423 spp.).

The problem of taxonomic inequality between native and non-native species may be solved by using the biogeographical approach ([14]; see also Fig 1), where the same species are studied in their native and non-native area. A disadvantage of this approach, however, is the often large environmental difference between the two areas that are compared. For example, a plant species introduced from a high-latitude into a low-latitude area may recruit more herbivorous insect species than it had in its native area simply because it was transported into an area with a richer overall biodiversity.

Hence, using only one of the two approaches may lead to incomplete or misleading results (see Discussion, below). Combining both approaches in a single study can provide more robust results. However, only two studies employing such a combined approach have been published [15,16].

In this study we test the basic prediction of the ERH in above-ground plant—phytophagous insect systems, combining both the community and biogeographical approach reciprocally. We studied plants occurring in two different areas, viz. the Netherlands and Japan. As Europe and East Asia have a long history of reciprocal plant introductions [17], most plant species involved were either native to the Netherlands and non-native to Japan, or vice versa. All non-native species were established in the non-native area, and, in many cases, naturalised and/or invasive (in some cases, the introduced species were ornamentals and may have gone through a phase of artificial selection for appearance—we are unable to say whether this may have affected their defence against herbivores). If enemy release had been a factor in their establishment and spread, we would expect to find a signal of this in the degree by which they are presently impacted by phytophagous insects. Four aspects of the phytophagous insect communities were measured, namely insect species richness, insect abundance, insect dry weight, and degree of herbivory. We studied whether non-native plants (or plants growing in their non-native area) contain fewer phytophagous insects than native plants (or plants growing in their native area). Furthermore, we used our data to compare the community and the biogeographical approaches and the results of both approaches combined to indicate the weaknesses of only using one approach.

## Methods

### Data collection

No specific permits were required for the work described here. The samples were taken outside of protected areas and no endangered or protected species were collected. Phytophagous insects (from here on simply referred to as “insects”) were collected on native and non-native plants in two areas: Haren, province of Groningen, the Netherlands (53º10’N 06º36’E) and Sendai, Miyagi prefecture, Japan (38º16’N 140º52’E). In total, 47 plant species were sampled (Table 1), which could be divided into four groups: (1) plant species native to the Netherlands and non-native to Japan, or vice versa (14 spp.), (2) plant species non-native to both areas (5 spp.), (3) plant species studied only in the Netherlands; either native or non-native (9 spp.), and (4) plant species studied only in Japan; either native or non-native (19 spp.). This set-up enabled us to use both approaches reciprocally, by comparing native and non-native plants growing in the Netherlands and in Japan (community approach) and by comparing plants occurring in their native and non-native habitat, both in the Netherlands and Japan (biogeographical approach) (Fig 1).

**Table 1:** The 47 plant species used in this study.

Plant species	Plant origin	Studied in NL	Studied in Japan	Growth form	Comm. appr.^1^	Biogeo. appr.^1^	Herbi-vory^2^
*Acer palmatum*	Japan	*	*	Tree	*	*	*
*Amelanchier sp.*	N. America	*	*	Shrub	*	*	*
*Aucuba japonica*	Japan	*	*	Shrub	*	*	*
*Berberis thunbergii*	Japan	*	*	Shrub	*		*
*Betula pendula*	Netherlands	*		Tree	*		*
*Buddleja davidii*	China	*	*	Shrub	*		*
*Calycanthus floridus*	N. America		*	Shrub	*		
*Camellia japonica*	Japan		*	Shrub	*		*
*Carpinus betulus*	Netherlands	*		Tree	*		*
*Cercis chinensis*	East-Asia		*	Shrub	*		
*Cornus controversa*	Japan	*		Tree	*		
*Cornus officinalis*	Japan		*	Shrub	*		
*Corylopsis spicata*	Japan	*	*	Shrub	*	*	*
*Cunninghamia lanceolata*	East-Asia		*	Tree	*		
*Cytisus scoparius*	Netherlands	*	*	Shrub	*		*
*Davidia incolucrata*	China		*	Tree	*		
*Edgeworthia chrysantha*	Japan		*	Shrub	*		
*Fagus sylvatica*	Netherlands	*		Tree	*		*
*Fallopia japonica*	Japan	*	*	Herb	*	*	
*Forsythia suspensa*	Netherlands		*	Shrub	*		
*Ginko biloba*	China	*	*	Tree	*		*
*Hydrangea macrophylla*	Japan	*	*	Shrub	*	*	*
*Larix kaempferi*	Japan	*	*	Tree	*	*	*
*Liriodendron tulipifera*	N. America		*	Tree	*		*
*Lonicera japonica*	Japan	*	*	Vine	*	*	*
*Magnolia kobus*	Japan	*	*	Tree	*	*	*
*Metasequoia glyptostroboides*	Netherlands		*	Tree	*		
*Osmanthus fragrans*	Japan		*	Shrub	*		
*Parthenocissus quinquefolia*	N. America	*	*	Vine	*		*
*Parthenocissus tricuspidata*	Japan	*	*	Vine	*		*
*Picea abies*	Netherlands		*	Tree	*		
*Podocarpus macrophyllus*	Japan		*	Tree	*		
*Poncirus trifoliata*	East-Asia		*	Shrub	*		
*Populus alba*	Netherlands		*	Tree	*		
*Prunus serotina*	N. America	*		Tree	*		
*Rhamnus frangula*	Netherlands	*		Shrub	*		*
*Robinia pseudoacacia*	N. America	*	*	Tree	*		*
*Rosmarinus officinalis*	Netherlands		*	Shrub	*		
*Rubus fruticosus*	Netherlands	*		Vine	*		*
*Rubus phoenicolasius*	Japan	*		Vine	*		
*Rumex acetosa*	Netherlands	*	*	Herb	*	*	
*Skimmia japonica*	Japan	*	*	Shrub	*	*	*
*Sorbus aucuparia*	Netherlands	*		Tree	*		*
*Sorbus commixta*	Japan		*	Tree	*		*
*Spiraea japonica*	Japan		*	Shrub	*		*
*Taxodium distichum*	N. America		*	Tree	*		
*Viburnum plicatum*	Japan	*	*	Shrub	*	*	

For each species origin and growth form are indicated, the area in which the species was studied, the approach used, and whether data were collected on the level of herbivory.

^1^ Plant species for which the data were collected on insect diversity and insect load

^2^ Plant species for which the data were collected on the level of herbivory, all using the community approach

We tested for differences between native and non-native plant species (community approach) and between plants growing in their native and non-native area (biogeographical approach) in number of insect species, number of insect individuals, dry weight of all insects, and level of herbivory. Assuming that greater species richness per se also translates as greater negative impact on the plants (through more diversified damage and more ways to evade plant defenses [18,19]), all four categories of data would correlate with the negative impact of natural enemies. The first three categories of data were collected simultaneously in both areas between late April and early August, 2010. The data on the level of herbivory were collected non-simultaneously, during several consecutive days. Insect herbivory is highly seasonal, especially with regard to bursts of larval maturation in, e.g., Lepidoptera and Coleoptera [20]. For this reason, a biogeographical comparison of the level of herbivory in Japan and the Netherlands would not have been appropriate. Therefore, we considered this category only in the context of the community approach. Insect collection took place in forests, parks, and gardens. In both the Netherlands and Japan the same two standardised methods were used to sample insects. Insects were collected by shaking the branches of the plant for 10 s above a beating sheet of 1 m^2^ or by sweeping an insect net with a diameter of 50 cm for 10 s through the branches of the plant. All individuals of the same plant species were sampled with the same method. The collecting methods were rehearsed and standardised (in the Netherlands) by all collectors beforehand.

Collected insects were stored in 70% ethanol. Insects from nine taxonomic groups (Table 2) were collected. For four taxonomic groups, experts were found to identify the species (Table 2, indicated by *). For all collected insects the insect load was determined in two ways: by counting the number of insect individuals per sample, and by determining their dry weight. The levels of herbivory were estimated for 17 plant species in the Netherlands and 16 in Japan, using other plant individuals than the ones we sampled insects from. We did this as follows. We first randomly collected ten leaves from a plant and counted the leaves with insect damage. This was repeated for 5–27 individuals per plant species (mean: 11.9). Then, the mean leaf area was determined per plant species, by scanning on average 42 (range: 10–190) leaves per plant species using a flatbed scanner, and calculating the surface area using the software Lafore [21]. Finally, a herbivory index was devised, as [proportion leaves damaged / mean leaf area * 10,000].

**Table 2:** Number of individuals collected per taxonomic group.

Insect groups collected	Number of Individuals
Coleoptera: Curculionoidae (weevils)*	91
Hemiptera: Auchenorrhyncha (mainly cicadas)	108
Hemiptera: Heteroptera (mainly plant-, leaf-, and grass bugs)*	22
Hemiptera: Psyllidae (psyllids)*	88
Hemiptera: Sternorrhyncha (mainly aphids)	1410
Hymenoptera: Tenthredinidae (sawfly-larvae)	39
Orthoptera: Tettigoniidae (bush-crickets)*	4
Lepidoptera (caterpillars)	149
Thysanoptera (thrips)	124

For all groups, the number of insect individuals and dry weight of all insects was established; all individuals of groups indicated by * were identified for establishing the number of insect species.

### Statistical analysis

All data were analysed using a linear mixed model (library: lme4) in the statistical program R [22]. The significance of variables was tested by comparing models with and without variables using Chi-square statistics, and non-significant variables (critical value α = 0.05) were removed using a backwards removal method based on lowest *P*-values. Data collected using the community approach were analysed separately from data collected using the biogeographical approach.

#### Community approach

Four sets of data, collected using the community approach, were analysed: the number of insect species, number of insect individuals, dry weight of all insects and herbivory level (Table 3). For the first three sets of data the model included four fixed factors (species status [native / non-native], area [NL / JP], collection date, and height of the plant sampled), the interaction between species status and area, and the random factor plant species. The data on number of insect species and number of insect individuals were square-root transformed to normalise the data (square-root transformation was chosen because it yielded the best fit with a normal distribution). For the data set on herbivory level the model included three fixed factors (species status, area, and height of the plant sampled), the interaction between species status and area, and the random factor plant species. Date of collection was not included because the data for herbivory level were collected within a few days.

**Table 3:** Overview of the statistical results for the community and biogeographical approach.

COMMUNITY APPROACH			BIOGEOGRAPHICAL APPROACH		
**Number of insect species**					
	*χ* ^2^	*P*		*χ* ^2^	*P*
Species status	20.37	<0.0001	Species status	-	-
Area	4.939	0.0263	Origin	-	-
Date of collection	16.34	<0.0001	Date of collection	18.251	<0.0001
Plant height	9.272	0.0023	Plant height	43.976	<0.0001
Species status * Area	0.370	0.5429	Species status * Origin	14.157	0.0002
**Number of insect individuals**					
	*χ* ^2^	*P*		*χ* ^2^	*P*
Species status	4.770	0.0290	Species status	-	-
Area	10.027	0.0015	Origin	-	-
Date of collection	0.461	0.4972	Date of collection	0.962	0.3266
Plant height	0.141	0.7072	Plant height	0.672	0.4123
Species status * Area	0.028	0.8664	Species status * Origin	9.294	0.0023
**Dry weight of all insects**					
	*χ* ^2^	*P*		*χ* ^2^	*P*
Species status	-	-	Species status	1.281	0.2577
Area	-	-	Origin	1.151	0.2834
Date of collection	1.741	0.1870	Date of collection	0.425	0.5146
Plant height	0.814	0.3669	Plant height	1.275	0.2589
Species status * Area	4.072	0.0436	Species status * Origin	0.360	0.5484
**Level of herbivory**					
	*χ* ^2^	*P*			
Species status	-	-			
Area	-	-			
Leaf surface	8.382				
Species status * Area	8.838	0.0030			

Variables are explained in detail in the methods.

#### Biogeographical approach

Three sets of data, collected using the biogeographical approach, were analysed: the number of insect species, number of insect individuals and dry weight of all insects (Table 3). For all sets of data the model included four fixed factors (area status, origin, collection date, and height of the plant sampled), the interaction between species status and origin, and the random factor plant species. The data on number of insect species and number of insect individuals were square-root transformed to normalise the data. It must be noted that the biogeographical approach is limited by the fact that only two plant species from the Netherlands were available for this (against 11 from Japan).

## Results

A total of 502 individual plants (NL: 214, JP: 288) of 47 species (NL: 28, JP: 38) were sampled (Table 1) and 2,035 phytophagous insects were collected (Table 2). The results of the statistical analyses are shown in Table 3. All data shown in the text and in figures are averages ± SE and are calculated from all data combined.

### Community approach

Using the community approach, native and non-native plants were compared within the same area, either the Netherlands or Japan.

#### Number of insect species

**Fig 2:**
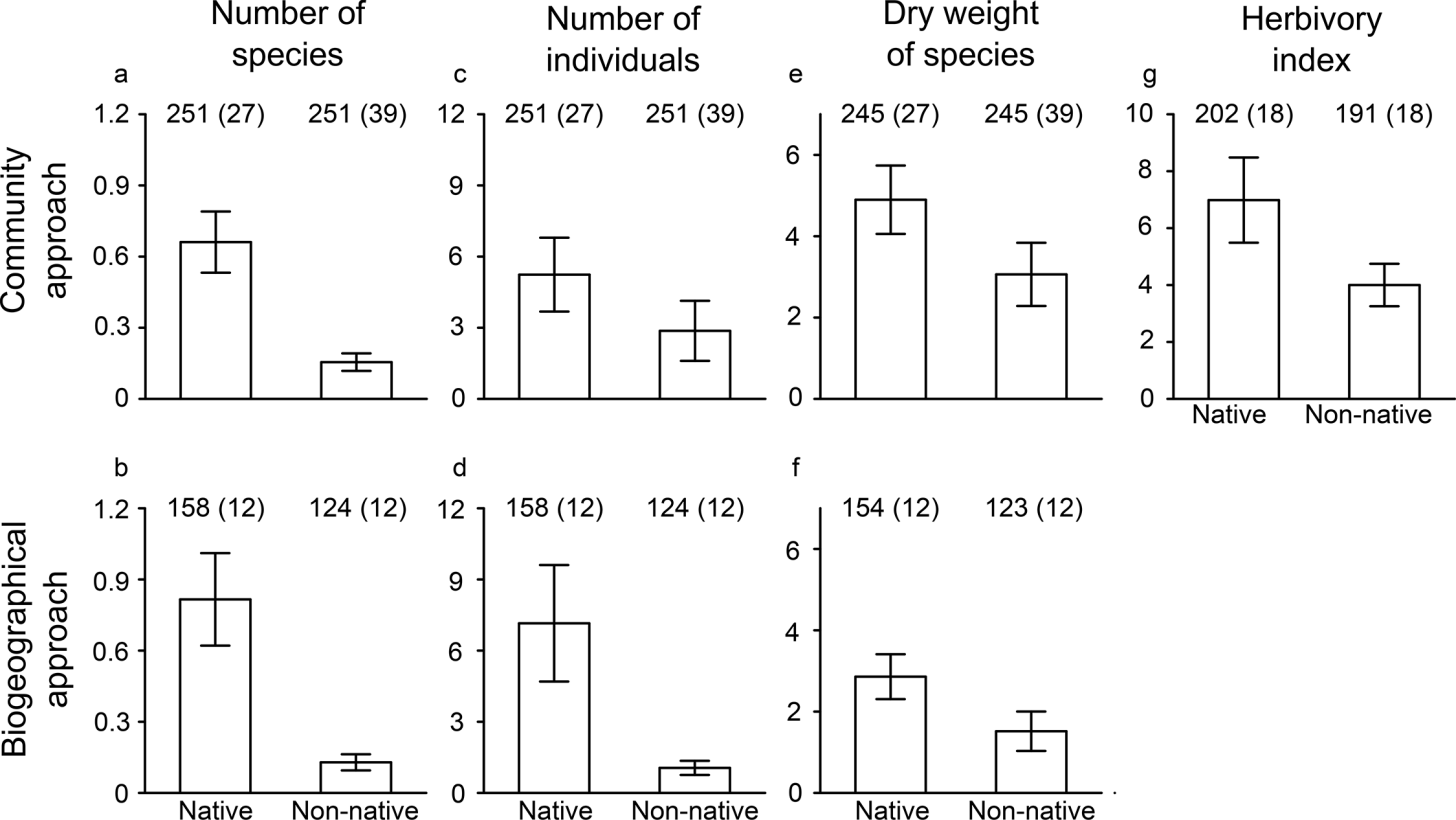
Comparison of insect abundance using the community and biogeographical approach. Shown are the number (mean ± 1 SE) of insect species (a, b), number of insect individuals (c, d), dry weight of all individuals (e, f) and level of herbivory (g) for native and non-native plant species (community approach) and plants growing in their native and non-native area (biogeographical approach). Sample sizes are shown: n = number of plants sampled (with number of species in parentheses). All figures are based on the untransformed data.

The number of insect species was more than four times higher on native plants than on non-native plants (0.66 ± 0.13 and 0.16 ± 0.04, resp.; Fig 2A). This difference was slightly greater in Japan (0.70 ± 0.17 and 0.13 ± 0.07, resp.) than in the Netherlands (0.57 ± 0.17 and 0.17 ± 0.04, resp.) and overall, the number of insect species was higher in Japan than in the Netherlands. Furthermore, there was an effect of date of collection and plant height; the number of insect species increased during the season, and with plant height.

#### Number of insect individuals

The number of insect individuals was two times higher on native than on non-native plants (5.24 ± 1.56 and 2.87 ± 1.27, resp.; Fig 2C). This difference was greater in the Netherlands (1.96 ± 0.34 and 0.65 ± 0.10, resp.) than in Japan (6.66 ± 2.22 and 5.58 ± 2.80, resp.) and overall, the number of insect individuals was higher in Japan than in the Netherlands. There was no effect of date of collection or plant height.

#### Dry weight of all insects

On average, the dry weight of all insects collected was c. 1.5 times higher on native than on non-native plants (4.90 mg ± 0.84 and 3.06 mg ± 0.78, resp.; Fig 2E). The interaction between species status and area was significant. In the Netherlands, the difference in dry weight was three times higher on native than on non-native plants (8.18 mg ± 2.21 and 2.57 mg ± 0.61, resp.). In Japan, there was no difference between native and non-native plants (3.45 mg ± 0.70 and 3.67 mg ± 1.57, resp.). There was no effect of date of collection or plant height.

#### Level of herbivory

Overall, the herbivory index was about one-third higher on native plants than on non-native plants (6.98 ± 1.50 and 4.01 ± 0.75, resp.; Fig 2G). There was an interaction between species status and area. In the Netherlands, the herbivory index was much higher on both native and non-native plants (14.15 ± 3.57 and 4.88 ± 0.92, resp.) than in Japan (2.19 ± 0.33 and 0.61 ± 0.08, resp.), but in both areas the level of herbivory was higher on native than on non-native plants.

### Biogeographical approach

Using the biogeographical approach, plants were compared when growing in their native and their non-native area, either the Netherlands or Japan.

#### Number of insect species

Overall, the number of insect species was six times higher on plants growing in their native area than in their non-native area (0.82 ± 0.20 vs. 0.13 ± 0.03; Fig 2B). There was an interaction between area status and origin. For plants originating from the Netherlands, the difference in the number of insect species between the native and the non-native area was much larger (1.32 ± 0.45 and 0.00 ± 0.00, resp.) than for plants originating from Japan (0.72 ± 0.22 and 0.16 ± 0.04, resp.), but in both cases there were more insect species on plants growing in their native area. Furthermore, there was an effect of date of collection and plant height; the number of insect species increased during the season and with plant height.

#### Number of insect individuals

The number of insect individuals was almost seven times higher on plants growing in their native area than in their non-native area (7.15 ± 2.46 vs. 1.06 ± 0.3; Fig 2D). There was an interaction between area status and origin. For plants originating from the Netherlands, there was no difference in the number of insect individuals between their native and non-native area (2.96 ± 0.73 and 3.73 ± 1.53, resp.). For plants originating from Japan, however, this difference was very large (7.94 ± 2.91 vs. 0.48 ± 0.09, resp.). There was no effect of collection date or plant height.

#### Dry weight of all insects

Overall, there was no significant difference in the dry weight of all insects between plants growing in their native and their non-native area (resp. 2.86 ± 0.55 and 1.52 ± 0.49). There was also no effect of collection date or plant height.

## Discussion

Using the community approach, we investigated four measures for herbivory, viz. species richness, abundance, and dry weight of phytophagous insects (the latter two being a measure for herbivore load), and the level of herbivory. Overall, these four different tests show the same result: on native plants the insect species richness, insect load and levels of herbivory are higher. All four tests jointly provide a clear indication that non-native plants indeed escape from their enemies, in this case herbivorous insects, as the ERH predicts [11].

Using the biogeographical approach, however, our results were less conclusive. In line with the prediction under the ERH, the insect species richness was higher on plants when growing in their native area. Furthermore, the insect abundance was higher on plants from Japan growing in their native area, but not on plants from the Netherlands when growing in the Netherlands. Dry weight of all insects did not differ between plants growing in their native and their non-native area. Thus, the data collected using the biogeographical approach do provide some support for the ERH, but they are not conclusive. This might be due to the rather small number (12 out of 47) of plant species available for this approach, and the fact that most of these species were from Japan, rather than from the Netherlands.

The lack of full reciprocal support from both the community and the biogeographic approaches reveal the vulnerability of using only one of the two approaches, which has been the predominant manner of addressing this issue. With reference to Fig 1, when comparing a trait between native and non-native species, four different methods can be used: (1) the community approach in area A; (2) the community approach in area B; (3) the biogeographical approach using species originating from area A; (4) the biogeographical approach using species originating from area B. If the trait studied were only influenced by the status of the species (native vs. non-native), all four methods would show an effect of status. However, in practice it is likely that environmental differences between the areas studied, and non-random phylogenetic distances between the species selected can greatly influence the trait values measured. We combined the community and biogeographical approach to obtain a more robust manner of addressing the ERH, and the results from our study illustrate this.

In our study, insect **species richness** was mainly affected by the status of the plant species: in the Netherlands as well as in Japan the insect species richness was higher on native plants; and Japanese as well as Dutch plants supported more insect species when growing in their native than their non-native habitat. Hence, for the trait of insect species richness, both approaches reinforced one another.

Insect **abundance** (one measure of herbivore load) showed a pattern suggesting a combined effect of status and area. Using the community approach, the insect abundance was affected by the (native/non-native) status of the species, but comparing Dutch species in the native (Netherlands) and non-native area (Japan) shows no effect of status, while comparing Japanese species show a very large effect of status. This leads to two conclusions, namely (i) that the insect abundance is higher on native plants than on non-native plants, and (ii) that overall insect abundance on all plants is higher in Japan than in the Netherlands.

For insect **dry weight** (the second measure of herbivore load), the results are less clear. Using the community approach, in the Netherlands the dry weight is higher on native than on non-native plants, while in Japan there is no difference. Using the biogeographical approach, also no differences were found between plants growing in their native and their non-native area. Apparently, other factors (e.g., taxonomic differences between Dutch and Japanese phytophagous insects) are influencing the insects’ dry weight.

The **level of herbivory** was only tested using the community approach, and herbivory levels were higher on native than on non-native plants both in the Netherlands and in Japan.

In conclusion, our results provide support for the prediction of the ERH that insect herbivory on non-native plants is reduced. (We note that, to test the ERH more fully, studying the plant demographic effects of enemy release is also required—this falls outside the scope of this study, however.) Two other studies employing combined community and biogeographic approaches obtained similar results. Norghauer et al. [16] found levels of herbivory in introduced *Swietenia macrophylla* in Dominica that were 3–6 times lower than on native trees, and 4–14 times lower than on *S*. *macrophylla* in the native range. Southwood et al. [15] compared a number of introduced and native tree species in Britain and South Africa and found that introduced trees carried lower species diversities for one particular herbivore guild (leaf-chewers).

By using a combined approach, it is possible to qualify the responses among the various measures of herbivory, and to reveal how the application of either approach in isolation could produce misleading results. We therefore advocate that more studies aiming to test the ERH use a combination of the community and biogeographic approach.

## References

[1] Agrawal AA, Kotanen PM (2003) Herbivores and the success of exotic plants: a phylogenetically controlled experiment. Ecol. Lett. 6: 712–715.

[2] Agrawal AA, Kotanen PM, Mitchell CE, Power AG, Godsoe W, Klironomos J (2005) Enemy release? An experiment with congeneric plant pairs and diverse above- and belowground enemies. Ecology 86: 2979–2989. 16227219

[3] Blossey B, Nötzold R (1995) Evolution of increased competitive ability in invasive nonindigenous plants: a hypothesis. J. Ecol. 83: 887–889.

[4] Colautti R, Ricciardi A, Grigorovich I, MacIsaac H (2004) Is invasion success explained by the enemy release hypothesis? Ecol. Lett. 7: 721–733.

[5] Gerber E, Krebs C, Murrell C, Moretti M, Rocklin R, Schaffner U (2008) Exotic invasive knotweeds (*Fallopia* spp.) negatively affect native plant and invertebrate assemblages in European riparian habitats. Biol. Cons. 141: 646–654.

[6] Heywood VH, Drake JA, Moony HA, di Castri F, Groves RH, Kruger FJ et al (1989) Patterns, extents and modes of invasions by terrestrial plants In: Drake JA et al, editors. Biological invasions, a global perspective. Hoboken: Wiley pp. 31–60.

[7] Hierro JL, Maron JL, Callaway RM (2005) A biogeographical approach to plant invasions: the importance of studying exotics in their introduced and native range. J. Ecol. 93: 5–15.

[8] Jones CG, Lawton JH (1991) Plant chemistry and insect species richness of British umbellifers. J. Anim. Ecol. 60: 767–777.

[9] Joshi J, Vrieling K (2005) The enemy release and EICA hypothesis revisited: incorporating the fundamental difference between specialist and generalist herbivores. Ecol. Lett. 8: 704–714.

[10] Keane RM, Crawley MJ (2002) Exotic plant invasions and the enemy release hypothesis. Trends Ecol. Evol. 17: 164–170.

[11] Kennedy CEJ, Southwood TRE (1984) The number of species of insects associated with British trees: a re-analysis. J. Anim. Ecol. 53: 455–478.

[12] Lehsten V (2005) Functional analysis and modelling of vegetation. PhD thesis, Carl von Ossietzky University, Oldenburg, Germany.

[13] Liu H, Stiling P, Pemberton RW, Peña J (2006) Insect herbivore faunal diversity among invasive, non-invasive and native *Eugenia* species: implications for the enemy release hypothesis. Florida Ent. 89: 475–484.

[14] Mitchell CE, Power AG (2003) Release of invasive plants from fungal and viral pathogens. Nature 421: 625–627. 12571594 10.1038/nature01317

[15] Norghauer JM, Martin AR, Mycroft EE, James A, Thomas SC (2011) Island invasion by a threatened tree species: evidence for natural enemy release of mahogany (*Swietenia macrophylla*) on Dominica, Lesser Antilles. PLoS ONE 6: e18790 doi: 10.1371/journal.pone.0018790 21533206 10.1371/journal.pone.0018790 PMC3076449

[16] Pimentel D (2001) Economic and environmental impacts of invasive species and their management. Pesticides and You 21: 10–11.

[17] R Development Core Team (2010) R: A language and environment for statistical computing Vienna: R Foundation for Statistical Computing.

[18] Southwood TRE, Moran VC, Kennedy CEJ (1982) The richness, abundance and biomass of the arthropod communities on trees. J. Anim. Ecol. 51: 635–649.

[19] Torchin ME, Lafferty KD, Dobson AP, McKenzie VJ, Kuris AM (2003) Introduced species and their missing parasites. Nature 421: 628–630. 12571595 10.1038/nature01346

[20] Van der Weijden W, Leewis RJ, Bol P (2007) Biological globalisation: Bio-invasions and Their Impacts on Nature, the Economy, and Public Health. Zeist: KNNV.

[21] Visser ME, Both C (2005) Shifts in phenology due to global climate change: the need for a yardstick. Proc. R. Soc. B 272: 2561–2569. 16321776 10.1098/rspb.2005.3356 PMC1559974

[22] Williamson MH (1996) Biological invasions. London: Chapman and Hall.

